# Expression of NKX2.2 in Non-Ewing Tumors With Round Cell Morphology

**DOI:** 10.7759/cureus.50704

**Published:** 2023-12-18

**Authors:** Saad M Saeed, Usman Hassan, Mudassar Hussain, Sajid Mushtaq, Sheeba Ishtiaq

**Affiliations:** 1 Pathology, Shaukat Khanum Memorial Cancer Hospital and Research Centre, Lahore, PAK; 2 Pathology, Ghulab Devi Teaching Hospital, Lahore, PAK

**Keywords:** wilms tumor, synovial sarcoma, rhabdomyosarcoma, neuroblastoma, lymphoblastic lymphoma, nkx2.2, ewing sarcoma

## Abstract

Background

Round cell sarcomas pose diagnostic challenges due to overlapping histopathological features, necessitating precise immunohistochemical markers for accurate categorization. NKX2.2 has emerged as a sensitive diagnostic tool, particularly in Ewing sarcoma. This study extends this understanding to various round-cell sarcomas, shedding light on the potential diagnostic utility of NKX2.2 beyond its established role. The nuanced exploration of NKX2.2 expression aims to enhance diagnostic strategies, prognostic assessments, and therapeutic developments in the landscape of sarcoma research.

Methodology

Cases were retrieved from the surgical pathology and consultation files of Shaukat Khanum Memorial Cancer Hospital and Research Center, Lahore, Pakistan. Representative hematoxylin and eosin-stained slides of six different types of already confirmed tumors, including lymphoblastic lymphoma, neuroblastoma, rhabdomyosarcoma, synovial sarcoma, Wilms tumor, and Ewing sarcoma, were reviewed by a panel of pathologists. Immunohistochemistry, utilizing a rabbit anti-NKX2.2 monoclonal antibody, was performed on formalin-fixed paraffin-embedded tissue sections. The presence of NKX2.2 was defined as moderate or high nuclear immunoreactivity in at least 5% of cells.

Results

The histopathological examination revealed characteristic features in each sarcoma subtype, aligning with established diagnostic criteria. In Lymphoblastic lymphoma, T-cell lineage was confirmed through TdT expression, while the atypical finding of focal NKX 2.2 expression hinted at genetic diversity. Neuroblastoma exhibited the expected salt and pepper chromatin pattern, with NKX 2.2 expression raising questions about its prognostic significance. Rhabdomyosarcoma presented primitive cells expressing desmin, and NKX 2.2 focal expression echoed previous subtype-associated studies. Synovial sarcoma displayed both monophasic and biphasic growth patterns and TLE1 expression, with NKX 2.2 variation suggesting tumor heterogeneity. In Wilms tumor, the characteristic WT1 expression was observed, while NKX2.2's absence reaffirmed its irrelevance in this context. Ewing sarcoma displayed the anticipated homogenous cell population, strong NKX2.2 expression, and CD99 positivity across various sites. Furthermore, age and gender impact on this range of sarcomas found no significant relation with an expression of NKX2.2.

Conclusion

In conclusion, the diverse expression profiles of diagnostic markers discovered in this study, particularly the atypical expression of NKX2.2 beyond its established role in Ewing sarcoma, signify a significant advancement. This unique finding accentuates the potential diagnostic importance of NKX2.2 in various sarcomas, presenting a novel dimension to our understanding of these malignancies.

## Introduction

Ewing sarcoma is an aggressive neoplasm and most often occurs in children and young adults [1]. The diagnosis has proved difficult for clinicians and pathologists because it belongs to the category of “small round blue cell tumor.” Many other sarcomas are also identified under this umbrella, all of which have almost similar morphologic characteristics [2].

The tumor has high aggressiveness and tends to develop in bone but may be seen even within soft tissue. Upon examination under a microscope, Ewing sarcoma cells appear small, round, and blue - which is one of the main diagnostic features for this sarcoma [3]. Nevertheless, its morphological similarities with other tumors create confusion in diagnosis. In addition, several other types of a tumor have confusing round cell morphology, thus making the differentiation even more difficult while looking through the microscope [4]. Some of them are different types of sarcomas like rhabdomyosarcoma which is derived from skeletal muscles and synovial sarcoma which affects soft tissues and joints [5]. Furthermore, this appears just like non-mesenchymal neoplasms such as lymphoblastic lymphoma, which is a common lymphoid neoplasm that mostly affects children and teenagers [6].

The similarity of Ewing sarcoma to pediatric tumors like Wilms tumor and neuroblastoma makes diagnosis difficult as these originate from the kidney and adrenal gland, respectively [7-9]. There is also a need for comprehensive diagnosis, even though the cells appear as blue as that of Ewing sarcomas, they are indeed diverse malignancies, making it important to utilize various methods such as immunohistochemistry, molecular tests as well and clinical presentation to identify [10,11]. However, among this clinical entrapment, it is important to acknowledge the historical and modern essence of this problem. These small round blue cell tumors are considered difficult ones based on previous studies and diagnostic experience. Such entities are difficult to distinguish neatly, requiring multiple modal diagnostics of histology, immunohistochemistry, and genotypes [12].

However, traditional diagnostic tools sometimes fail to decisively differentiate between these entities, maintaining uncertainty and likely influencing clinical decision-making. These problems have been highlighted with no real specific and sensitive marker differentiating the Ewing sarcoma from its histogenic doubles [13]. In this regard, one of the key markers examined is NKX2.2. It is a maker that has been proven to be very specific and sensitive for the diagnosis and detection of Ewing sarcoma, especially where the EWSR1-FLI1 translocation exists [14]. This marker has proven useful in diagnosing classic Ewing sarcoma cases, becoming an indispensable instrument of the pathologist's case kit. Nevertheless, the critical issue is that most research on NKX2.2 expression in Ewing sarcoma has mainly been directed toward classic Ewing sarcoma and EWSR1-FLI1 rearrangements. Consequently, a knowledge gap exists in which the diagnostic efficacy of NKX2.2 in other round tumors with blue cell morphology has not been explored [15]. Therefore, we have limited our knowledge about NKX2.2's suitability as a diagnostic marker and rely on some special forms of tumors among these complex tumors [16].

In order to improve the diagnosis of small round blue cell tumors, this study investigated more closely the specifics of this diagnostic problem, considered the details of molecular complexities, and studied the possible diagnostics for NKX2.2 on different types of tumor entities. This will provide a basis for specific and evidence-based approaches to managing these sarcomas to improve patient outcomes. This study's objective is to fill the gap in a large-scale exploration of NKX2.2 expression profiling other than classical Ewing sarcoma and to open up a new horizon for this marker in malignancy with small round cell origin. At the same time, this study aimed to thoroughly investigate the diagnostic role of NKX 2.2 in non-Ewing round cell tumors.

## Materials and methods

Already confirmed cases were retrieved from the surgical pathology and consultation files of Shaukat Khanum Memorial Cancer Hospital and Research Center, Pakistan. Representative hematoxylin and eosin-stained slides were reviewed. In total, whole-tissue sections of tumors were evaluated for expression of NKX2.2: 42 cases of lymphoblastic lymphoma, 47 cases of neuroblastoma, 65 cases of rhabdomyosarcoma, 25 cases of synovial sarcoma, 23 cases of Wilms tumor, and 231 cases of Ewing sarcoma.

Immunohistochemical staining of various antibodies including NKX2.2, CD99, TdT, synaptophysin, desmin, TLE1 & WT1 was studied. IHC was conducted on 4-mm thick formalin-fixed paraffin-embedded tissue sections after pressure cooker antigen retrieval (0.01 M citrate buffer, pH 6.0) for all the antibodies including rabbit anti- NKX2.2 (EP336) monoclonal antibody (Cell Marque Corporation, Rocklin, CA) [17,18], anti-CD99 (O13) mouse monoclonal primary antibody (Roche Diagnostics), anti-TdT (339-L-H) mouse monoclonal antibody (Leica Biosystems), anti-synaptophysin (299-L-CE) mouse monoclonal antibody (Leica Biosystems), anti-desmin (DE-R-11) mouse monoclonal antibody (Roche Diagnostics), anti-TLE1 (1F5) mouse monoclonal antibody (Cell Marque Corporation, Rocklin, CA) and anti-WT1 (6F-H2) mouse monoclonal antibody (Dako). Throughout the investigation, appropriate positive and negative external controls were applied. Positive controls include already diagnosed case of Ewing sarcoma for NKX2.2, pancreatic islet cells for CD99, normal thymus for TdT, already diagnosed neuroendocrine tumor for synaptophysin, the normal smooth muscle tissue of intestine for desmin, normal blood vessels lined by endothelial cells for TLE1 and fallopian tube for WT1. The strength of the staining (weak, moderate, or strong) and the proportion of cells with nuclear staining (0, 05%; 1+, 5%-25%; 2+, 25%-50%; 3+, 50%-75%; or 4+, 75%-100%) were used to evaluate the amount of immunoreactivity of NKX2.2. The presence of NKX2.2 was defined as moderate or high nuclear immunoreactivity in at least 5% of cells. While for other antibodies, i.e., CD99, TdT, synaptophysin, desmin, TLE1, and WT1 the results were recorded as positive or negative for the corresponding tumor. A panel of pathologists reviewed the slides using an advanced microscope (Olympus CX23).

## Results

**Figure 1:**
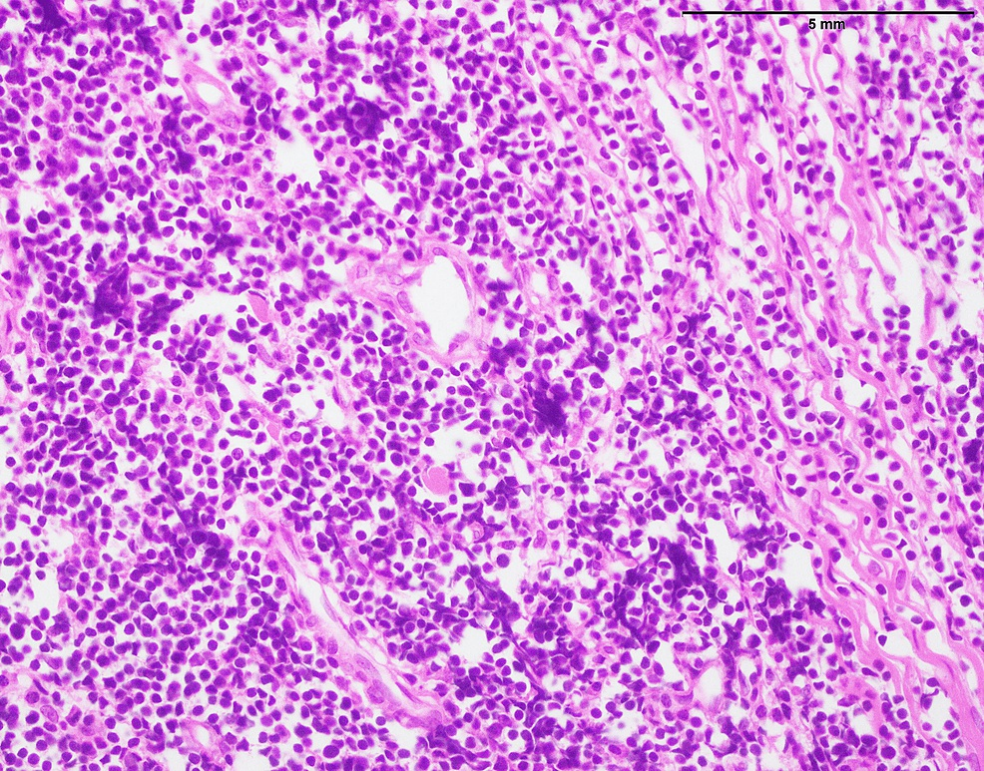
Lymphoblastic lymphoma: vertebral mass biopsy showing proliferation of small- to medium-sized cells with finely dispersed chromatin and inconspicuous nucleoli (400x).

**Figure 2:**
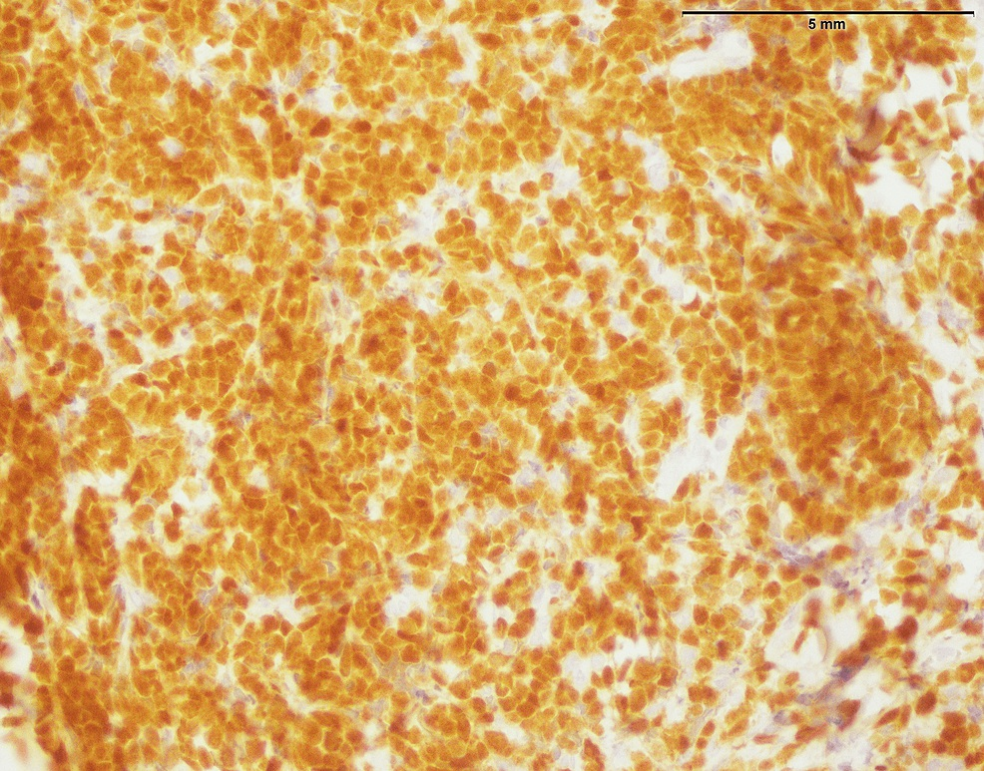
Lymphoblastic lymphoma: small- to medium-sized cells with strong positive expression of TdT (400x). TdT: Terminal deoxynucleotidyl transferase

**Figure 3:**
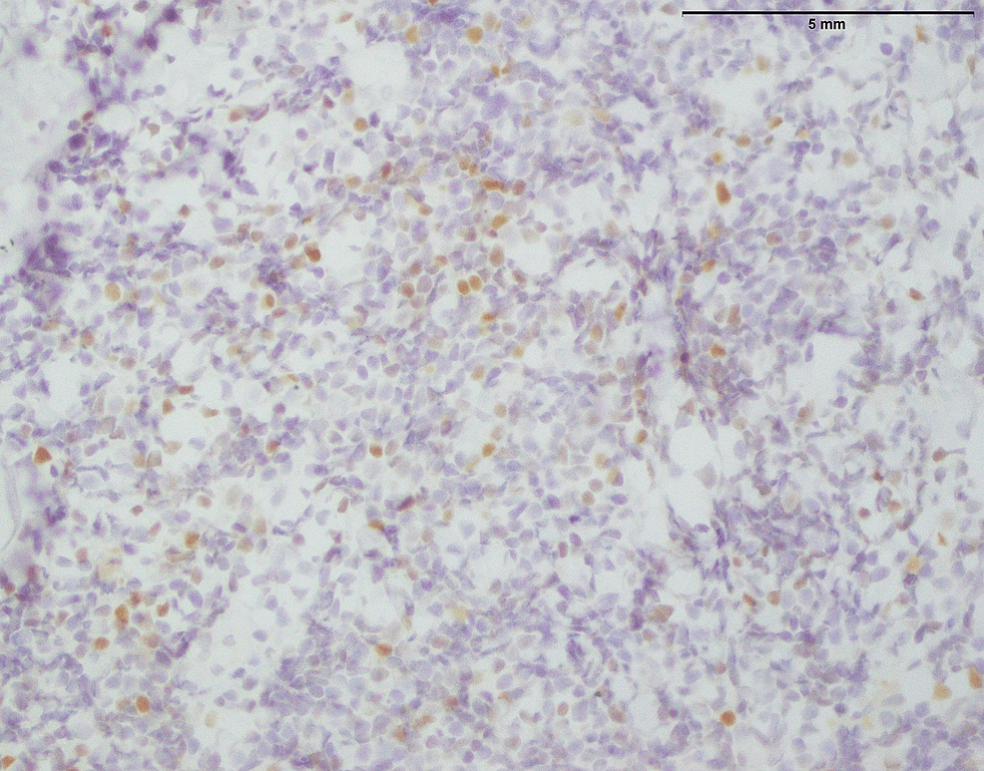
Lymphoblastic lymphoma: NKX 2.2 showing focal expression in few lymphoma cells (200x). _^NKX2.2: NK2 Homeobox 2^_

Forty-two already confirmed cases of lymphoblastic lymphoma were included, affecting 32 males and 10 females, with mean and median ages of 19 and 20, respectively. The anatomic distribution of lymphoblastic lymphoma included upper limb (n =3), lower limb (n =10), chest wall (n = 3), abdomen (n =8), mediastinum (n = 3), vertebra (n =3), inguinal region (n =2), lung (n =2), pleura (n=1), para-spinal area (n=1), liver (n =4), head and neck (n =1), and axilla (n =1). The lymphoblastic lymphoma's distinct histopathological characteristics include the presence of small to medium-sized cells with finely dispersed chromatin and inconspicuous nucleoli, reflecting their lymphoblastic nature as shown in Figure 1. This histological profile aligns with the diagnosis of T-cell lymphoblastic lymphoma, where TdT expression is a reliable marker for T-cell lineage, further confirming the lymphoma's identity (Figure 2). However, the focal and weak expression of NKX 2.2, an uncommon finding in this context, opens avenues for deeper exploration (Figure 3). It may imply genetic variations or cellular heterogeneity within the lymphoma, prompting the need for extensive molecular and genetic studies to fully grasp its clinical implications and potential impact on prognosis and treatment strategies.

**Figure 4:**
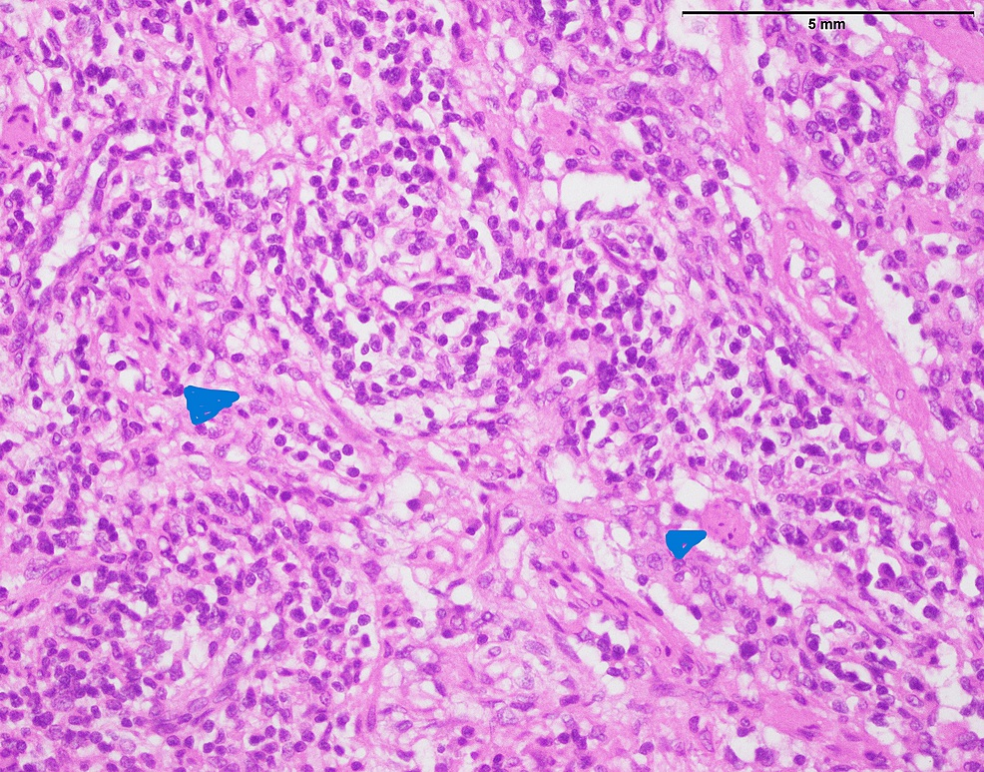
Neuroblastoma: biopsy from abdominal mass showing population of small- to medium-sized cells having salt and pepper chromatin pattern, scanty cytoplasm. Note the delicate eosinophilic neuropil (arrowhead) (400x).

Forty-seven neuroblastoma cases affected 33 males and 14 females, with mean and median ages of 10 and six, respectively. The anatomic distribution of neuroblastoma included abdomen (n=15), head and neck (n=9), kidney (n=6), liver (n=2), inguinal area (n=2), adrenal (n=4), back (n=4), retroperitoneum (n=3), pelvis (n=1), and lower limb (n=1). The histopathological examination of neuroblastoma reveals a distinct cellular profile characterized by a population of small to medium-sized cells displaying a “salt and pepper” chromatin pattern, indicative of a variable distribution of densely stained chromatin within the nucleus. In most cases, they exhibit scant cytoplasm, which is common in poorly differentiated and primitive neuroblastoma (Figure 4). However, fine eosinophilic neuropil was visible, which is a distinctive neuroblastoma feature expressing the nervous part of the tumor.

**Figure 5:**
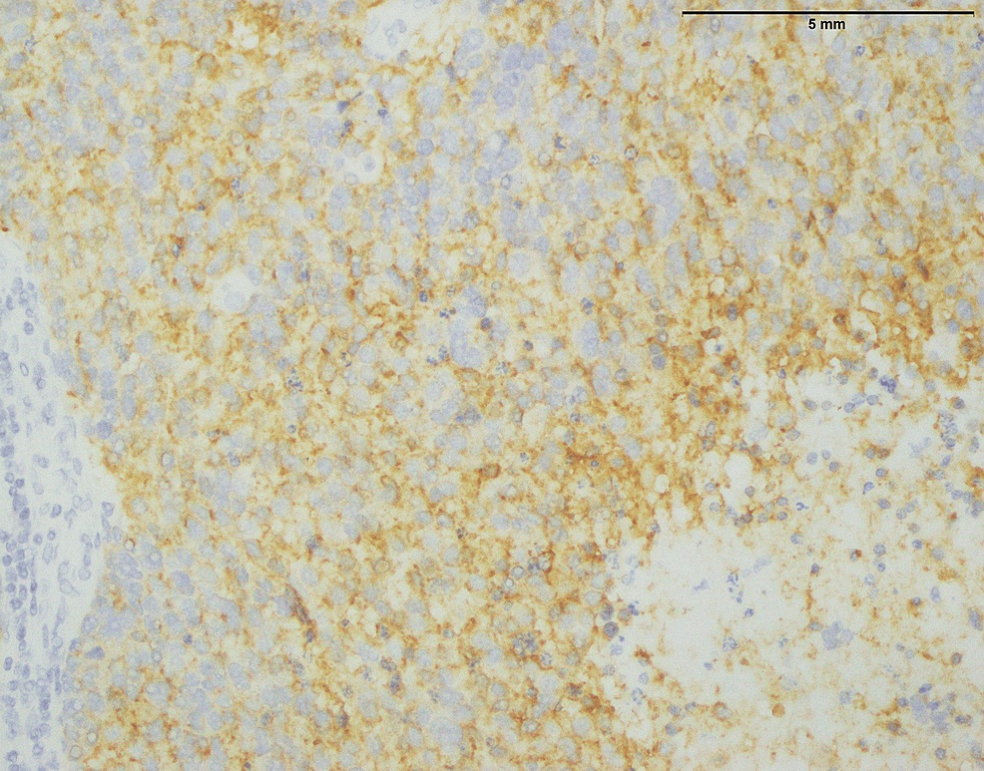
Neuroblastoma (abdominal mass): positive expression of synaptophysin in more than 90% tumor cells (400x).

**Figure 6:**
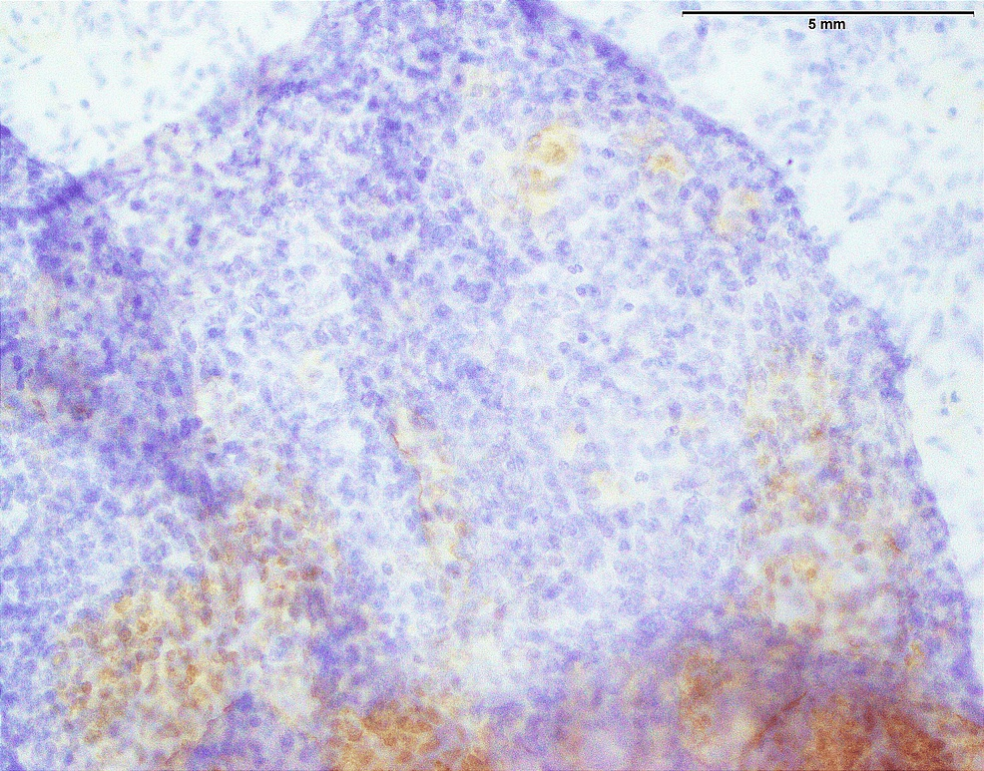
Neuroblastoma: photomicrograph showing majority of the cells are negative for NKX2.2; however, scattered focal expression can be seen in approximately 10% tumor cells (400x). NKX2.2: NK2 HOMEOBOX 2

Additional support for this diagnosis comes from immunohistochemical staining, which is shown in more than 90% of cells expressing synaptophysin a synaptic vesicle glycoprotein in neuroendocrine cells (Figure 5). The fact that this strong synaptophysin is indicative of the neuroendocrine characterization common among neuroblastomas. Besides, the fact that focal and low-level expression of NKX 2.2 is not a normal occurrence, indicates that there is some diversity within the tumor or rather specific gene traits (Figure 6). Further studies may require exploring this unusual NKX 2.2 expression and determining the clinical implication of the same. Together, these findings can improve diagnosis accuracy and may also be useful for prognosis and therapeutics for children with neuroblastoma.

**Figure 7:**
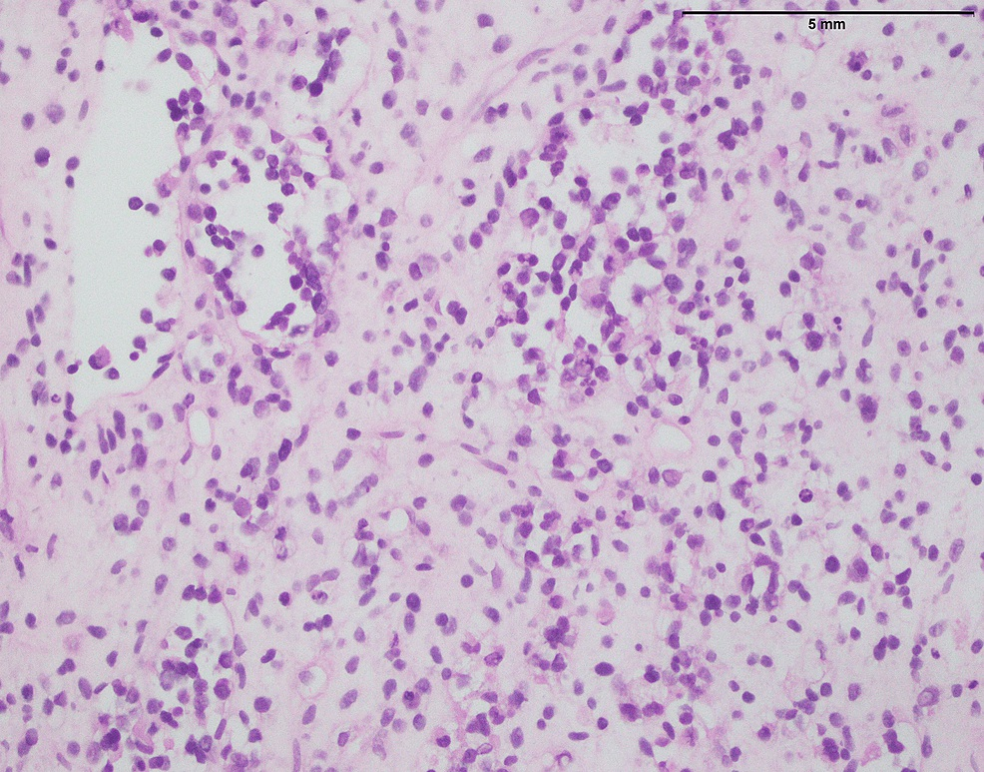
Rhabdomyosarcoma: tumor involving the orbit showing primitive looking small round cells with scanty cytoplasm, oval to rounded nuclei and inconspicuous nucleoli (400x).

**Figure 8:**
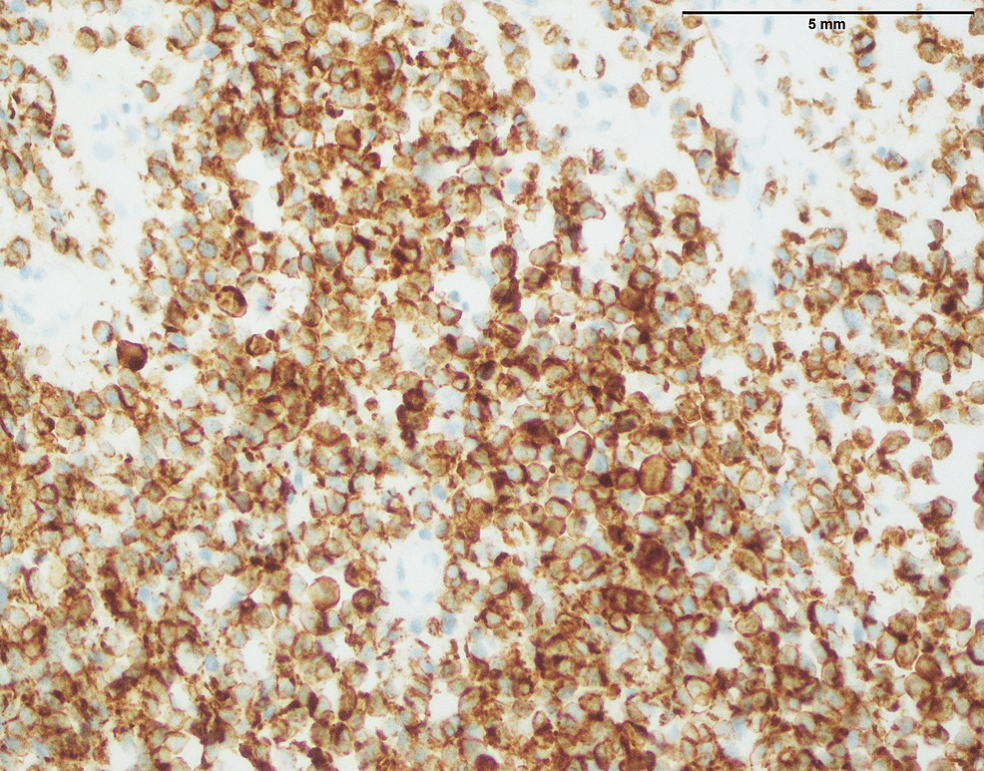
Rhabdomyosarcoma: desmin showing positive expression in majority of the tumor cells (400x).

Sixty-five rhabdomyosarcoma cases affected 40 males and 25 females, with a mean and median ages of 13 and 15, respectively. The anatomic distribution of rhabdomyosarcoma included head and neck (n=36), abdomen (n=7), upper limb (n=3), lower limb (n=5), testis (n=5), pelvis (n=3), back (n=2), chest wall (n=2), lungs (n=1), and anal verge (n=1). Histological examination of the rhabdomyosarcoma shows a unique histomorphology of round, small, immature-looking cells with very little cytoplasm. The cells have oval-rounded nuclei and inconspicuous nucleoli which suggest that these rhabdomyosarcomas are undifferentiated and primitive (Figure 7). Diagnosis confirmation was achieved through immunohistochemical staining. It is important that desmin was being positively expressed by tumor cells because this is a characteristic protein of muscular differentiation, providing evidence that these cells may be characterized as rhabdomyoblasts (Figure 8). This concurs with the diagnosis of rhabdomyosarcoma, a malignant tumor that arises from skeletal muscle precursors.

**Figure 9:**
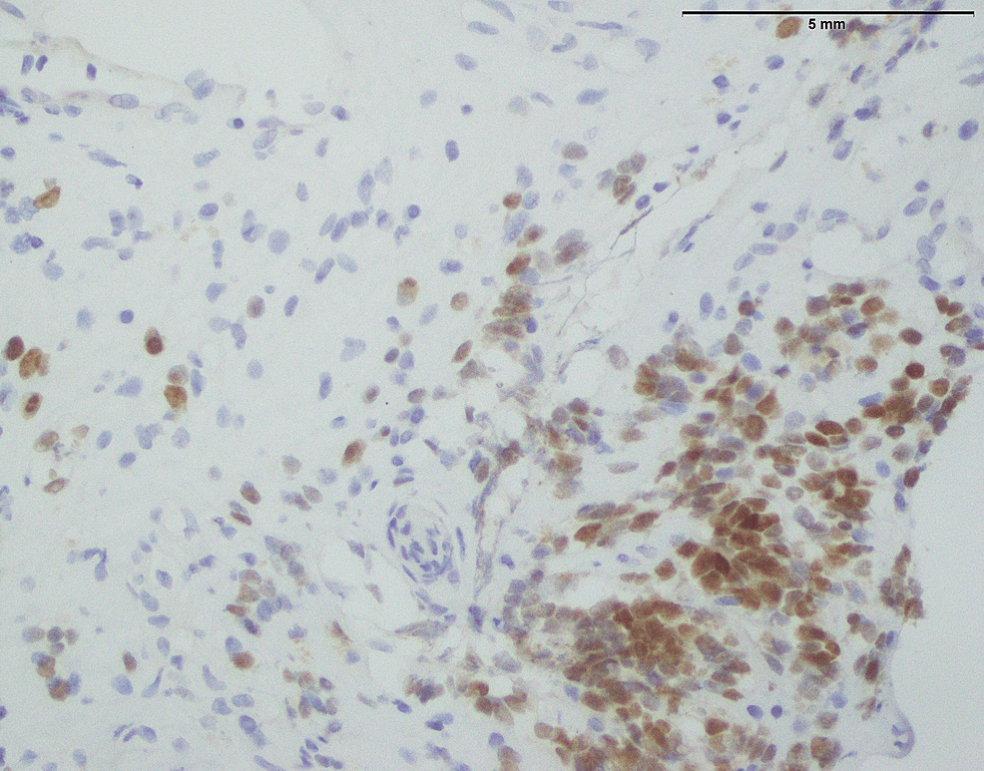
Rhabdomyosarcoma: focal NKX2.2 expression can be seen in few tumor cells (400x). NKX2.2: NK2 Homeobox 2

Further, the fact that the focal expression of NKX 2.2 was accompanied by moderate staining makes this observation intriguing (Figure 9). NKX 2.2 expression is not usually a hallmark of rhabdomyosarcoma. However, this uncharacteristic expression might suggest some degree of heterogeneity within the tumor or certain genetics in general. More studies are needed for further clarification regarding the clinical significance of the NKX 2.2 expression.

**Figure 10:**
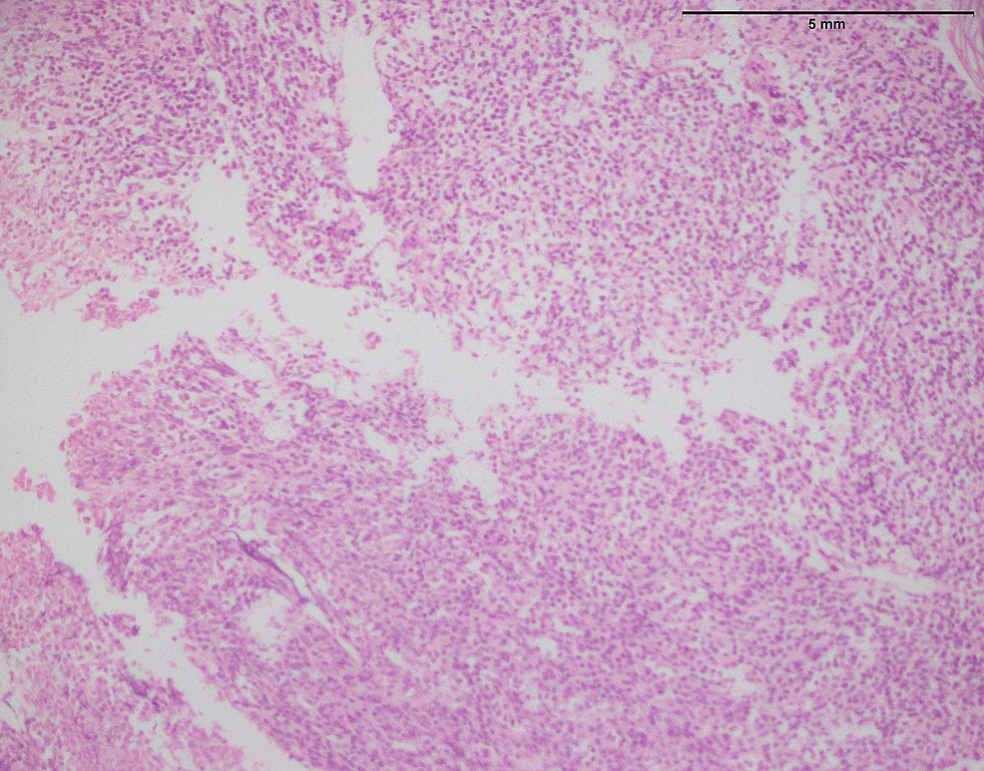
Synovial sarcoma: biopsy from the growth involving thigh having monophasic growth of round to spindled cells with scanty amphophilic cytoplasm, vesicular nuclei, and evenly dispersed chromatin (100x).

Twenty-five synovial sarcoma cases affected 12 males and 13 females, with a mean and median ages of 27 and 25, respectively. The anatomic distribution of synovial sarcoma included lower limb (n=13), head and neck (n=5), upper limb (n=2), kidney (n=2), and chest wall (n=1). In synovial sarcoma, both types of patterns (monophasic & biphasic) were seen. Cells are round or elongated cells with no discernable cell membranes little amphophilic cytoplasm, vesicular nuclei, and uniformly scattered chromatin (Figure 10).

**Figure 11:**
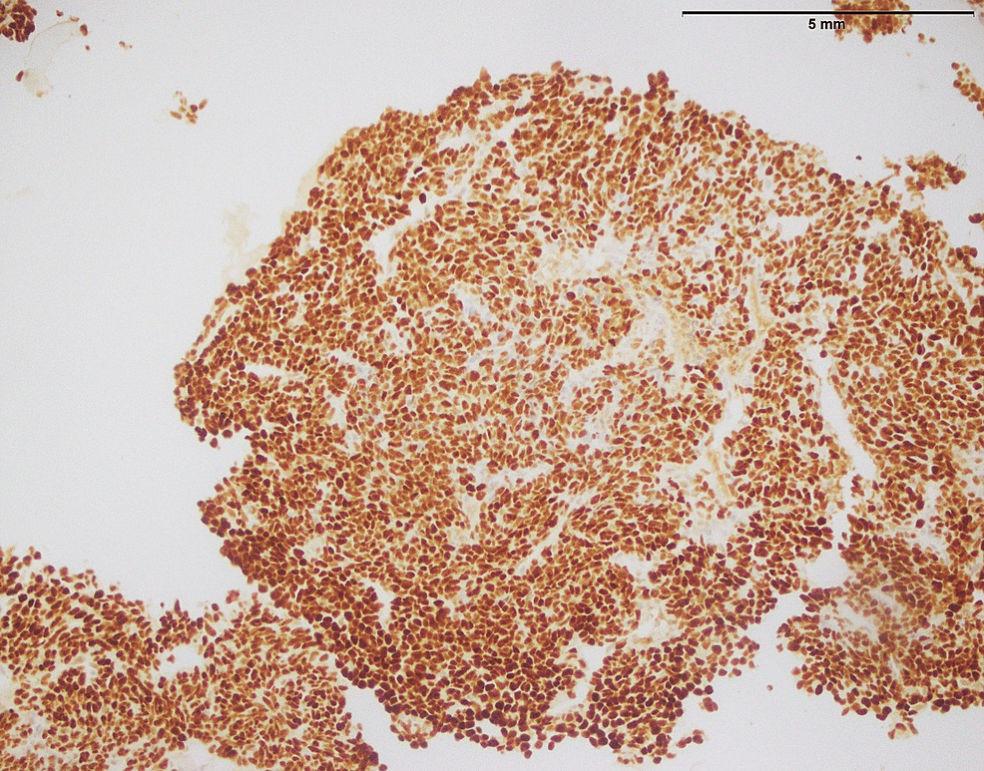
Synovial sarcoma: tumor cells showing positive expression of TLE1 (200x). TLE1: Transducin-like enhancer protein 1

**Figure 12:**
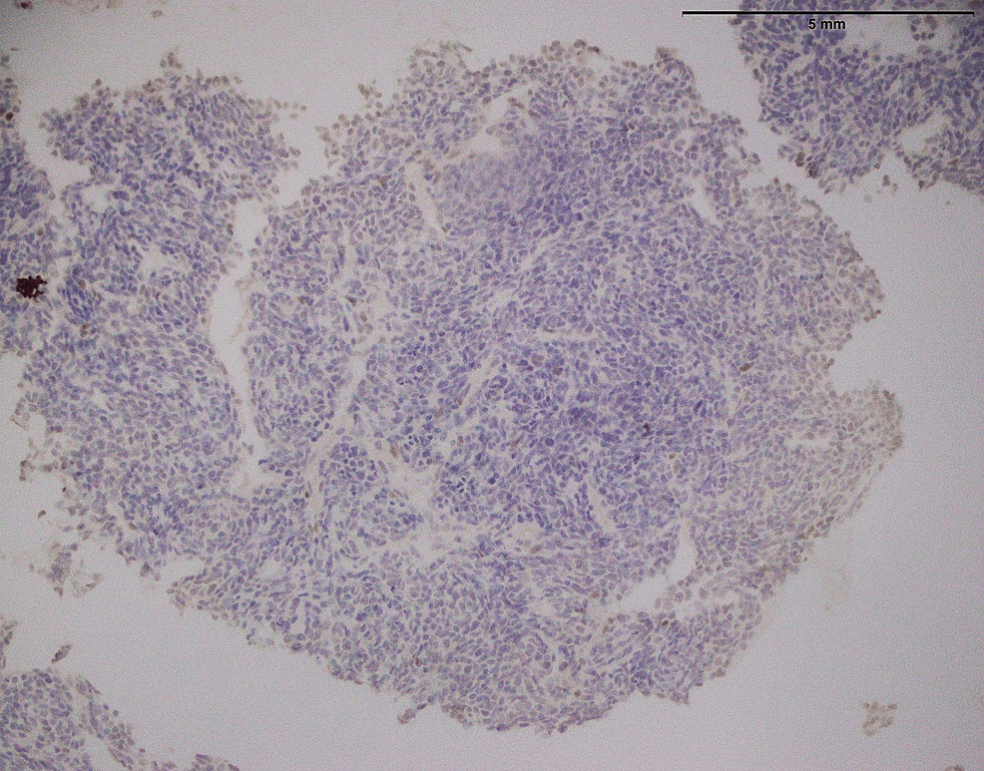
Synovial sarcoma showing focal expression of NKX2.2 in approximately 5%-10% tumor cells (200x). NKX2.2: NK2 Homeobox 2

The process of immunohistochemical staining serves as a pivotal component in confirming the diagnosis of synovial sarcoma. A noteworthy finding, in this case, was the strong positive expression of TLE1, which stands as a highly specific marker for synovial sarcoma (Figure 11). This robust TLE1 expression adheres to the established diagnostic criteria for synovial sarcoma, thereby enhancing the precision of the diagnosis. Additionally, the presence of focal expression of NKX 2.2 introduces an intriguing element to the results (Figure 12). While NKX 2.2 expression is not commonly associated with synovial sarcoma, this atypical observation suggests the possible existence of tumor heterogeneity or specific genetic attributes deserving further exploration. Investigating the clinical implications and underlying genetic factors related to NKX 2.2 expression in the context of synovial sarcoma is imperative and may provide valuable insights into the tumor's behavior and offer guidance for potential treatment strategies.

**Figure 13:**
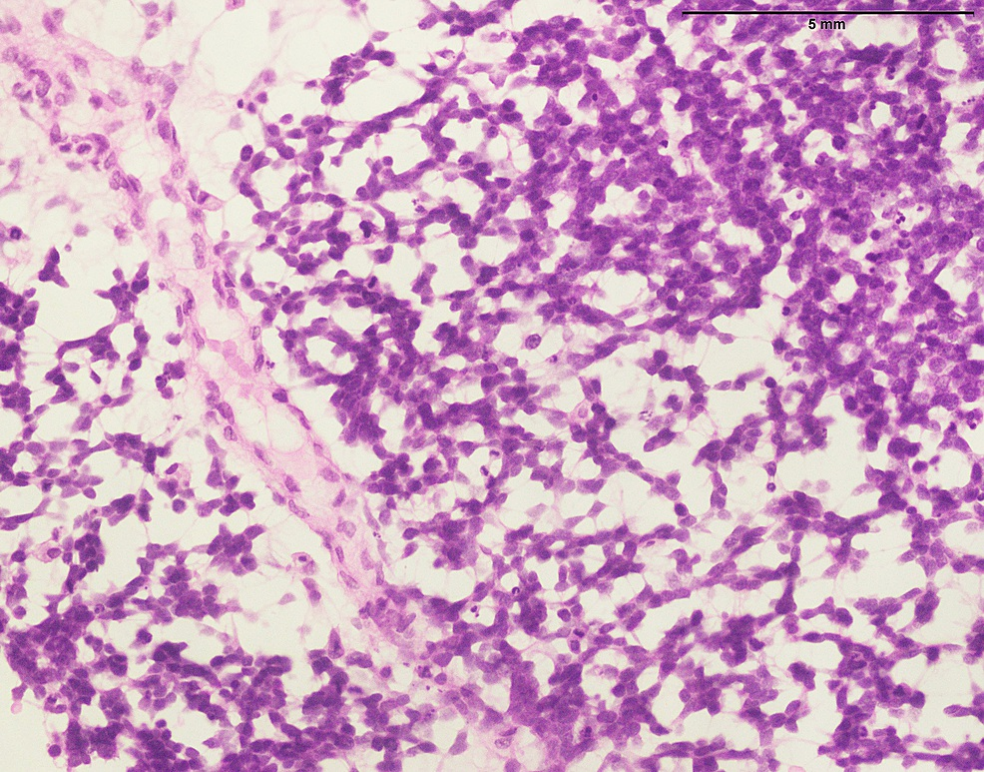
Wilms tumor: biopsy from kidney mass showing small to medium undifferentiated cells with regular looking nuclei and small nucleoli (400x).

**Figure 14:**
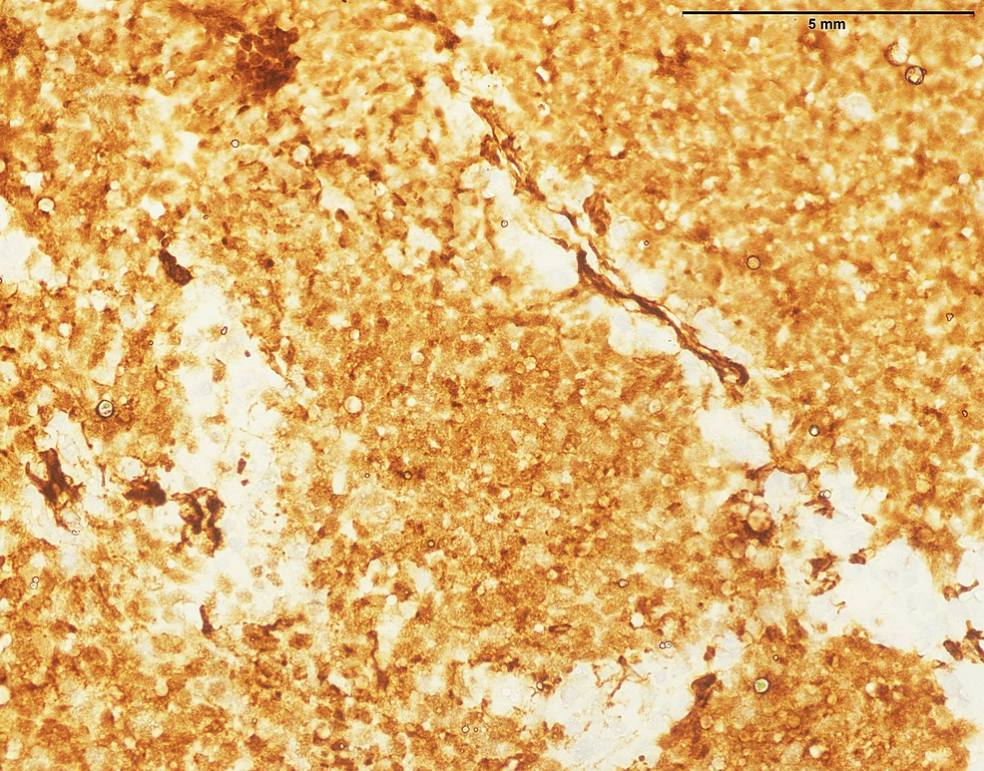
Wilms tumor: strong positive expression of WT1 (400x). WT1: Wilms tumor 1

Two cases of Wilms tumor, arising in the kidney affected 12 males and 11 females, with mean and median ages of six and five, respectively. A histopathologic specimen of the kidney mass from Wilms tumor would show undifferentiated cells of small to medium size with apparently normal-looking nuclei having minute-sized nucleoli (Figure 13). These indicate that the tumor occurs in primitive or primitive-like cells. Immunohistochemical analysis is very important here, and it is worth noting that the WT1 marker was strongly positively expressed (Figure 14). The high level of WT1 expression is in line with the characteristic pathological findings of Wilms tumor, and it represents a useful tool to identify this childhood renal neoplasm.

**Figure 15:**
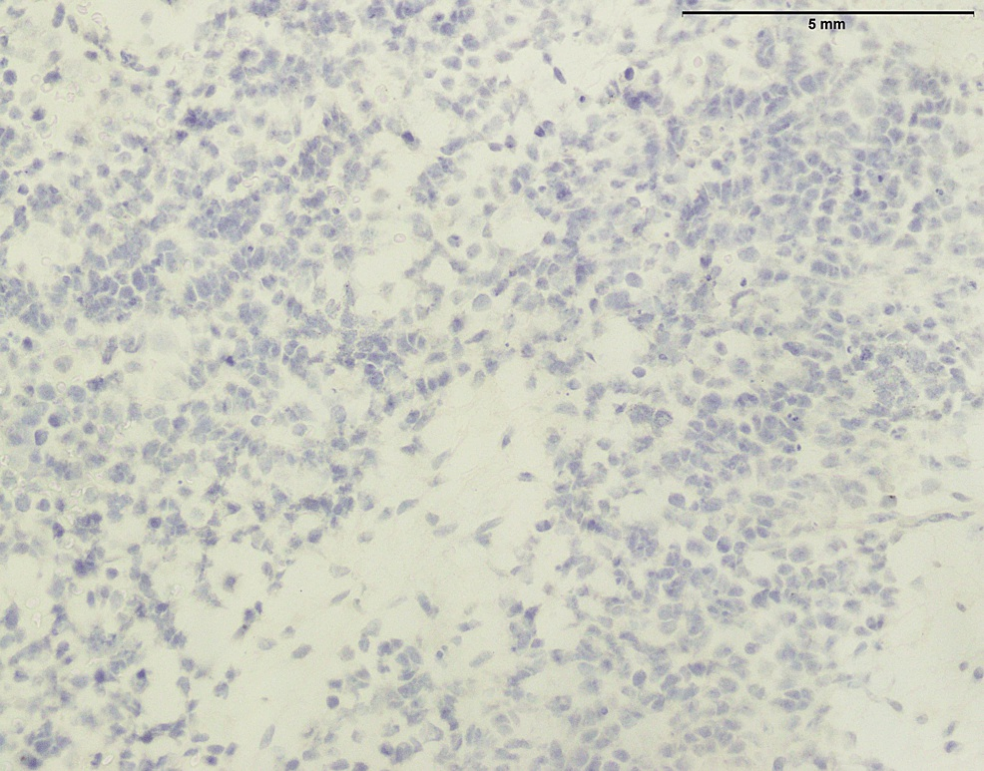
Wilms tumor: no NKX2.2 expression is seen (400x). NKX2.2: NK2 Homeobox 2

The lack of NKX 2.2 expression is quite significant since NKX 2.2 is largely unrelated to the Wilms tumor (Figure 15). Moreover, this immunohistochemical profile helps to confirm the correct diagnosis of the tumor and facilitates differential diagnosis with Ewing sarcoma. Therefore, detailed histological and immunohistochemical characterization is vital for accurate diagnosis of Wilms tumor and related effective treatment options.

**Figure 16:**
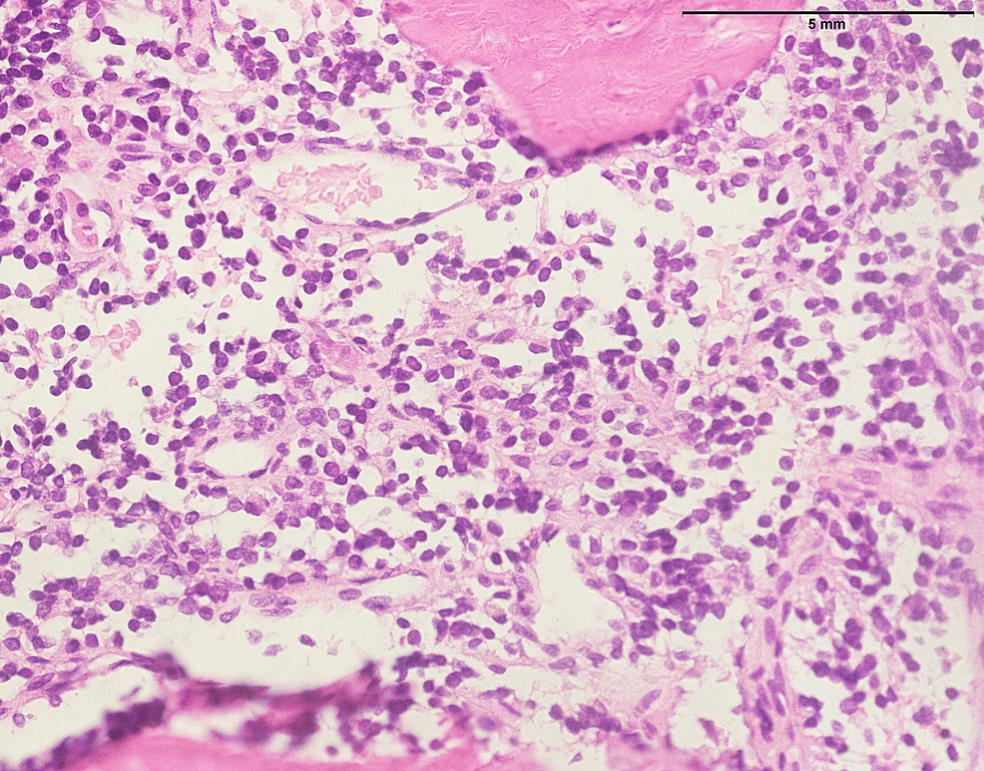
Ewing sarcoma: biopsy from a lower limb soft tissue mass involving the bone, showing uniform population of small round cells with rounded nuclei and fine stippled chromatin. Note the nucleoli are inconspicuous and cytoplasm is scanty (400x).

**Figure 17:**
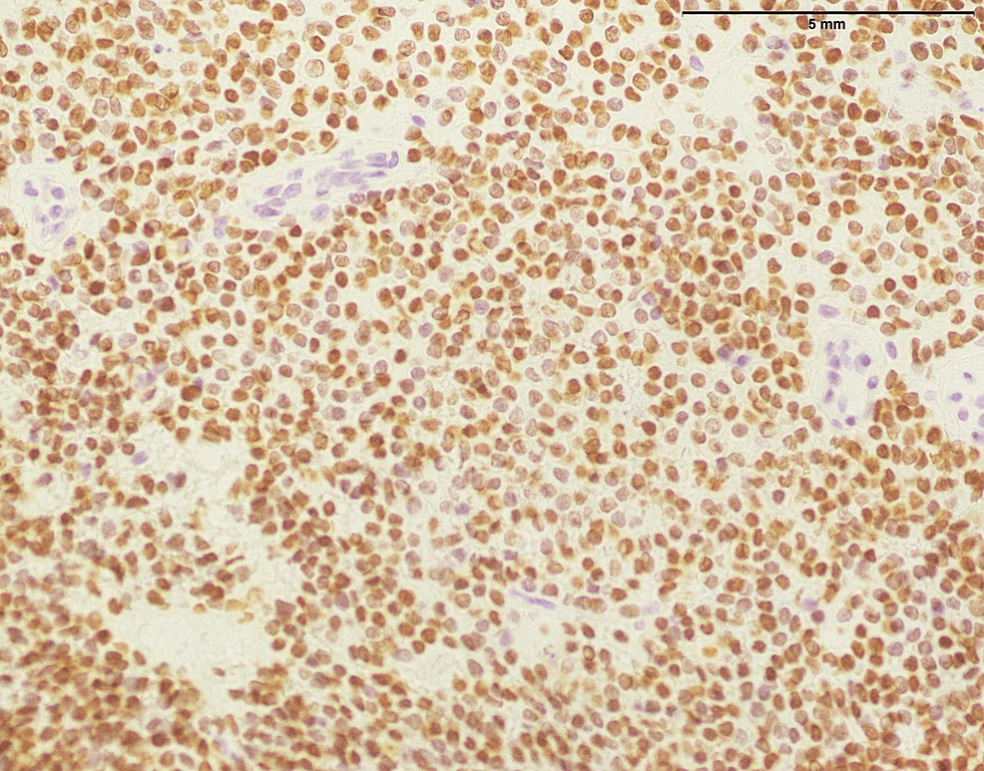
Ewing sarcoma: strong nuclear expression of NKX2.2 (400x). NKX2.2: NK2 Homeobox 2

**Figure 18:**
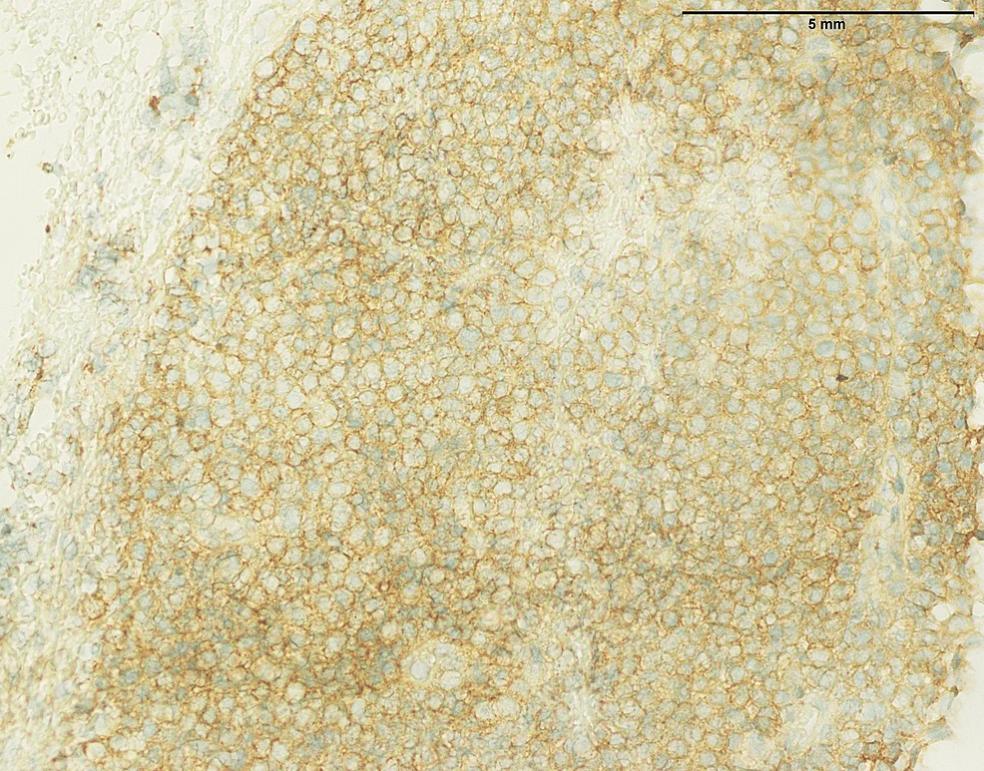
Ewing sarcoma: diffuse membranous expression of CD99 in tumor cells (400x). CD99: Cluster of differentiation 99

Two hundred thirty-one Ewing sarcoma cases affected 95 males and 136 females, with a mean and median age of 17 and 26, respectively. The anatomic distribution of Ewing sarcoma included lower limb (n=92), head and neck (n=25), upper limb (n=32), kidney (n=2), spine (n=24), pelvis (n=11), abdominal wall (n=6), retroperitoneum (n=3), breast ( n=1), testis (n=1), pancreas (n=1) lungs (n=2), and chest wall (n=32). Figure 16 shows a biopsy from a lower limb soft tissue mass involving the bone with characteristic features of Ewing sarcoma. The histopathological examination reveals a homogenous population of small round cells displaying rounded nuclei with finely stippled chromatin, underscoring the undifferentiated nature of the tumor. Notably, nucleoli are inconspicuous, and the cytoplasm is scanty, indicative of the primitive and aggressive nature of Ewing sarcoma. The immunohistochemical analysis contributes to the diagnostic profile, revealing a strong nuclear expression of NKX2.2, a transcription factor associated with neural differentiation (Figure 17). Furthermore, the tumor cells exhibit a diffuse membranous expression of CD99, a characteristic marker in Ewing sarcoma (Figure 18). These findings collectively contribute to the accurate identification of Ewing sarcoma, aiding in its distinction from other soft tissue and bone malignancies and providing essential information for precise diagnosis and tailored therapeutic strategies.

## Discussion

The research provides information on the diagnostic importance of NKX 2.2, particularly in the context of certain sarcoma subtypes. This study not only offers important insights into these individual tumors, but it also illustrates the larger context of histopathological and immunohistochemical features in round cell sarcomas. The findings of this study add greatly to our understanding of round-cell sarcomas by focusing on their histological and immunohistochemical features. The findings from diverse sarcomas give useful insights for improving diagnostic procedures and have consequences for therapy planning. It gained a thorough knowledge of the diagnostic and prognostic implications by reviewing these findings in the context of previously published studies. In the current study, lymphoblastic lymphoma has histological characteristics consistent with T-cell lymphoblastic lymphoma, which is confirmed by the confirmation of TdT expression. The unexpected observation of the localized expression of NKX 2.2 (Table 1) offers a nuanced layer, consistent with previous research demonstrating genetic variation within lymphoblastic lymphomas [19]. This highlights the importance of further exploring the molecular landscape in order to improve diagnostic techniques. Neuroblastoma's characteristic profile, which includes a salt and pepper chromatin pattern and the presence of a delicate eosinophilic neuropil, is consistent with recognized diagnostic criteria. The localized expression of NKX 2.2 (Table 1) adds complexity, echoing earlier results that point to possible prognostic importance and the need for more research into its function in risk stratification [20]. This emphasizes the dynamic nature of diagnostic markers and their developing consequences.

**Table 1:** Cases of different tumors with their positive percentage against NKX2.2 marker NKX2.2: NK2 Homeobox 2

Serial Number	Type of Tumor	Total Cases	NKX2.2 Expression (%)
1	Lymphoblastic lymphoma	42	1 (2.3)
2	Neuroblastoma	47	1 (2.1)
3	Rhabdomyosarcoma	65	1 (1.5)
4	Synovial sarcoma	25	3 (12)
5	Wilms tumor	23	0 (0)
6	Ewing sarcoma	231	231(100)

The primitive-looking tiny round cells expressing desmin in rhabdomyosarcoma match diagnostic criteria, but the localized expression of NKX 2.2 adds an intriguing feature (Table 1). A study by Hunk et al. linking NKX 2.2 expression to particular rhabdomyosarcoma subtypes emphasizes the necessity of subtype categorization for appropriate diagnosis and prognosis [21]. This emphasizes the expanding understanding of molecular markers in rhabdomyosarcomas. In synovial sarcoma, positive expression of TLE1, and localized weak expression of NKX 2.2 are common. The high level of TLE1 expression improves diagnostic accuracy however, the unusual NKX 2.2 expression shows tumor heterogeneity, which is consistent with previous research conducted by Ali et al. and Parez et al. on genetic variation in synovial sarcomas [22,23]. This highlights the importance of complex diagnostic interpretations when dealing with tumor subtype heterogeneity.

The typical histological characteristics and robust positive expression of WT1 in Wilms tumor match established diagnostic criteria. The lack of NKX2.2 expression demonstrates its insignificance as a marker in Wilms tumor. This emphasizes the significance of immunohistochemistry, particularly WT1 staining, incorrect diagnosis and differentiation of renal neoplasms. On the other hand, Ewing sarcoma has a homogeneous population of tiny round cells, robust nuclear expression of NKX2.2, and diffuse membrane expression of CD99, which is compatible with the diagnostic value of these markers, as described in earlier research [24,25]. The anatomic distribution of Ewing sarcoma in multiple places adds to the body of knowledge, underlining the necessity of these indicators in proper identification [26].

The discovery of NKX2.2 expression in other sarcomas, in addition to its documented sensitivity in Ewing sarcoma, is an unusual finding. While previous research has highlighted NKX2.2 as a diagnostic marker for Ewing sarcoma, its expression in different sarcomas expands its diagnostic value [27-29]. The newly discovered diagnostic capabilities of NKX2.2 over a larger spectrum of sarcomas has substantial clinical implications.

However, there are some limitations that need to be addressed notwithstanding the knowledge acquired from these findings because very less percentage of positive expression of NKX2.2 in round cell sarcomas is found except ewing sarcoma. The sample size was limited in terms of diversity, and it might not include a full description of the entire spectrum of round cell sarcoma due to the wide range of types, same reported in previous studies [29]. Furthermore, variations in tissue processing, staining process and subjective opinions of different pathologists would induce inherent bias as it already highlighted by Dunsten et al. [30]. However, this study is retrospective, and it affects the control over data collection, which in turn weakens the strength of the results obtained. Moreover, the lack of molecular analysis and genetic profiling limits more profound explanations of the reason why different sarcomas express NKX2.2 protein. Lastly, the fact that this was a single-centre study means that its findings cannot be easily applied to larger groups. Such limitations emphasize on multi-center studies having large cohorts and complete descriptions about expression of NKX2.2 in different round cell sarcomas.

## Conclusions

In conclusion, this study greatly increases our understanding of round-cell sarcomas, particularly in the setting of NKX2.2 expression. The unusual expression of NKX2.2 in diverse sarcomas highlights the importance of continuing research into the genetic and molecular heterogeneity within these malignancies. These findings have the potential to influence sarcoma research diagnostic techniques, prognosis assessment, and the development of targeted therapeutics. The changing landscape of diagnostic markers emphasizes the dynamic character of oncology, requiring a re-evaluation of previous paradigms and additional investigation of molecular fingerprints among distinct sarcoma subtypes.
